# The Consensus Definition of Bronchopulmonary Dysplasia Is an Adequate Predictor of Lung Function at Preschool Age

**DOI:** 10.3389/fped.2022.830035

**Published:** 2022-02-04

**Authors:** Segundo Rite, Carlos Martín de Vicente, Juan P. García-Iñiguez, María L. Couce, María P. Samper, Alicia Montaner, Carmen Ruiz de la Cuesta

**Affiliations:** ^1^Division of Neonatology, Department of Pediatrics, Miguel Servet University Hospital, Zaragoza, Spain; ^2^Department of Microbiology, Pediatrics, Radiology and Public Health, University of Zaragoza, Zaragoza, Spain; ^3^Division of Neumology, Department of Pediatrics, Miguel Servet University Hospital, Zaragoza, Spain; ^4^Division of Neonatology, University Clinical Hospital of Santiago, Santiago de Compostela, Spain; ^5^Department of Forensic Sciences, Pathology, Gynecology and Obstetrics, and Pediatrics, University of Santiago de Compostela, Santiago de Compostela, Spain; ^6^Health Research Institute of Santiago (IDIS), Santiago de Compostela, Spain; ^7^Division of Neonatology, Vall d'Hebron University Hospital, Barcelona, Spain

**Keywords:** bronchopulmonary dysplasia, lung function, preterm infant, spirometry, preschool age

## Abstract

**Background:**

Recent attempts to refine the definition bronchopulmonary dysplasia (BPD) have based its predictive capacity on respiratory outcome in the first 2 years of life, eliminating the pre-existing requirement of 28 days of oxygen therapy prior to 36 weeks postmenstrual age (PMA). The objective of this study was to assess the utility of the 2001 consensus definition in predicting impaired lung function at preschool age.

**Methods:**

This cohort study included children aged 4–6 years old who were born at gestational age (GA) <32 weeks or bodyweight <1500 g. Univariate and multivariate analyses were performed to assess differences in antenatal and neonatal variables between BPD and non-BPD children. All participants underwent incentive spirometry. Lung function parameters were contrasted with the Global Lung Function Initiative (GLI-2012) reference equations and, together with antenatal and neonatal variables, compared among the different subgroups (no BPD, mild BPD, and moderate-to-severe BPD). A multivariate model was generated to identify independent risk factors for impaired lung function.

**Results:**

GA, hemodynamically significant patent ductus arteriosus, and late sepsis were independent risk factors for the development of BPD. A total of 119 children underwent incentive spirometry. All lung function parameters were significantly altered relative to reference values. Greater impairment of lung function was observed in the mild BPD vs. the no BPD group (forced expiratory volume in the first 0.75 seconds [FEV_0.75_]: −1.18 ± 0.80 vs. −0.55 ± 1.13; *p* = 0.010), but no difference in forced vital capacity (FVC) was observed (−0.32 ± 0.90 vs. −0.18 ± 1; *p* = 0.534). The moderate-to-severe BPD group exhibited the most severe FEV_0.75_ reduction (FEV_0.75_: −2.63 ± 1.18 vs. −0.72 ± 1.08; *p* = 0.000) and was the only condition with FVC impairment (FVC: −1.82 ± 1.12 vs. −0.22 ± 0.87; *p* = 0.000). The multivariate analysis identified a diagnosis of moderate-to-severe BPD as an independent risk factor for lung function impairment.

**Conclusion:**

The 2001 consensus definition of BPD has adequate predictive capacity for lung function measured by spirometry at 4–6 years of age. Moderate-to-severe BPD was the best predictor of respiratory impairment. Children with mild BPD showed greater alteration of FEV_0.75_ than those without BPD.

## Introduction

In recent years there has been a significant decrease in mortality and morbidity among the most immature preterm infants. Bronchopulmonary dysplasia (BPD) is a chronic lung disease caused by multiple factors, including immaturity of the airway, that results in impaired lung growth, affecting both the airway and the pulmonary vessels (1). According to some reports, the incidence of this disease is not decreasing, but rather showing an upward trend (2). This condition is one of the most common sequelae of prematurity, and can lead to impairment of respiratory function that persists for life.

Since first described by Northway in 1967 (3), the diagnostic definition of BPD has undergone several changes (4, 5). Shennan's contribution (6) was the first that sought to predict death and respiratory morbidity in the first 2 years of life. The National Institute of Health (NIH) consensus definition, proposed in 2001, specified the requirement of supplemental oxygen for at least 28 days, and categorized BPD as mild, moderate, or severe at 36 weeks postmenstrual age (PMA) (7). Despite the consensus, debate arose over the following years about several aspects of the definition, including new modes of respiratory support (e.g., high-flow nasal cannulas) and potentially sub-optimal stratification according to disease severity (8). A recent workshop conducted at the Eunice Kennedy Shriver National Institute of Child Health and Human Development (NICHD) proposed further modifying the definition of BPD by incorporating the requirement of specific supplemental oxygen with oxygen saturation targeting, along with the mode of respiratory support and radiographic changes at 36 weeks PMA (9). However, the latter proposal, as well as that of Jensen et al. (10) based on their evidence-based approach, leave certain issues unresolved, especially because the Jensen study considers respiratory morbidity as a primary outcome exclusively during the first 2 years of life and does not consider long-term lung function outcomes.

In the present study we sought to evaluate the utility of the NIH consensus definition of BPD in predicting lung function compromise in preschool age children who underwent incentive spirometry.

## Methods

### Patients

This prospective study analyzed retrospectively collected data pertaining to a cohort of infants who were born between 2008 and 2011 in a level III hospital with a birth weight <1500 g and gestational age (GA) <32 weeks, and were identified in the neonatal follow-up unit between 2015 and 2016.

All children aged 4–6 years without congenital abnormalities or major neurological involvement were eligible to participate in the study. Exclusion criteria were as follows: severe cognitive impairment, cerebral palsy, history of post-haemorrhagic hydrocephalus, periventricular leukomalacia or additional comorbidity which may contribute to respiratory morbidity such as severe gastroesophageal reflux, etc. Episodic wheezing, acute viral bronchiolitis, and asthma were not considered criteria for exclusion.

Participants were seen by a pediatrician for physical examination, a respiratory questionnaire was completed by their parents, and a lung function test was conducted.

### Outcome Variables and Definitions

#### Antenatal and Neonatal Variables

Single or multiple gestation; premature rupture of membranes >18 h; clinical chorioamnionitis (11) (defined as the presence of uterine tenderness and/or purulent or foul-smelling amniotic fluid with any two of the following: antepartum temperature of 37.8°C or more, maternal tachycardia >120 beats/min, maternal leukocytosis >18,000 cells/mm^3^, or fetal tachycardia >160 beats/min); hypertensive disease of pregnancy; antenatal steroids; intrauterine growth restriction (12); sex; GA; birth weight; mode of delivery; clinical risk index for babies (CRIB) score (13); intubation in delivery room; mechanical ventilation modalities; need for invasive ventilation at 7 days of age; duration of invasive ventilation; need for inotropic support in first week of life; hemodynamically significant patent ductus arteriosus (PDA); need for surgical closure of PDA; late sepsis; BPD defined according to the NIH consensus definition (7) [supplemental oxygen required for at least 28 days, defined as *mild* [breathing room air at 36 weeks PMA or discharge], *moderate* (need for <30% oxygen at 36 weeks PMA or discharge), or *severe* (need for ≥30% oxygen and/or positive pressure at 36 weeks PMA or discharge)]. Cases of moderate and severe BPD were combined for the purpose of statistical analysis.

#### Respiratory Data Up to 4–6 Years of Age

Family history of allergy or asthma, parental smoking; history of atopic dermatitis and recurrent wheezing (three or more episodes in 6 months). At the time of the study, the presence of persistent recurrent wheezing was recorded.

#### Lung Function Parameters

Based on studies that support the use of forced spirometry as a method to assess lung function in preschool children (14, 15), incentive spirometry was carried out at 4–6 years of age (JaegerⓇ, MasterScreenⓇ, version 5.0). Each child performed up to eight maneuvers. The following parameters were recorded for each child: force expiratory time (FET); forced vital capacity (FVC); forced expiratory volume in the first second (FEV_1_) and first 0.75 s (FEV_0.75_); forced expiratory flow at 75% of FVC (FEF_75_); forced expiratory flow between 25 and 75% of FVC (FEF_25−75_); and FEV_1_/FVC – FEV_0.75_/FVC. The American Thoracic Society and European Respiratory Society quality control criteria (16), modified by Stanojevic (17), were applied. Z-scores were calculated for the spirometry parameters based on Global Lung Function Initiative 2012 (GLI-2012) equations (18). The GLI-2012 reference equations were recently validated in a cohort of healthy Spanish Caucasian preschool children aged 3–6 years (15). The GLI-2012 software enables calculation of the percentile and z-score for each lung function parameter for each child relative to the reference population. This allowed us to determine the percentage of children who were below the lower limit of normal (LLN) for each parameter, which corresponds to 5^th^ percentile or a z-score of −1.64.

FEV_0.75_ was preferred to FEV_1_ as FET can be <1 s in preschool children and FEV_0.75_ is also validated to evaluate lung function.

### Statistical Analysis

A sample size of 112 children was estimated to detect differences of at least 0.6 z-score points in lung function parameters between the groups with and without BPD (80% power and 95% confident interval), considering a BPD prevalence of 38% in our Neonatal Unit. The final study population with an adequate spirometric study fulfilled this condition.

Statistical analyses were performed with SPSS (version 21.0; IBM SPSS Statistics for Mac. Armonk, NY). Results are expressed as the mean (standard deviation, SD), median (interquartile range), or n (%) as appropriate. Univariate analysis was performed to assess the differences in antenatal, neonatal variables and subsequent diagnosis of recurrent wheezing during the first 4–6 years between subgroups with and without BPD. Differences were assessed using the Chi-squared or Fisher's exact test for categorical variables and the Student's *t*-test or Mann-Whitney U test for continuous variables. A logistic regression model was constructed, including variables for which significant results were obtained in the univariate analysis, using a backward stepwise strategy. In a second step, lung function parameters were contrasted with those of the GLI-2012 reference population (18). Given that all lung function parameters followed a normal distribution, a one-sample *t*-test was used for these analyses. Lung functions parameters were compared among the different subgroups and contrasted for all antenatal, neonatal, and respiratory variables using a Student's *t*-test. Multivariate multiple linear regression was performed for each lung function parameter applying variables for which significant results were obtained in the univariate analysis using a backward stepwise strategy.

In all tests a *p* < 0.05 was considered statistically significant.

## Results

**Figure 1:**
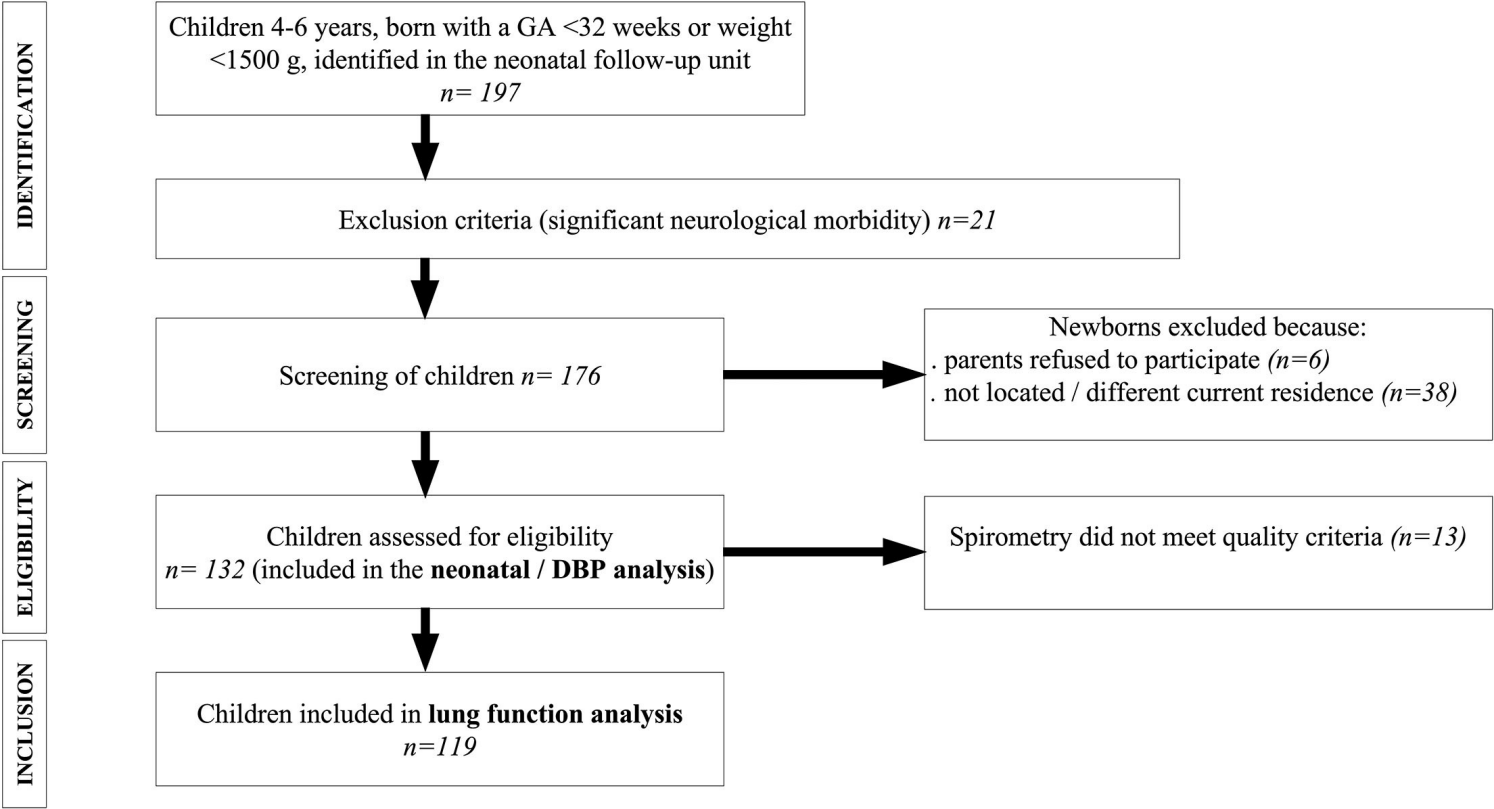
Flow chart of the study population. GA, gestational age; BPD, bronchopulmonary dysplasia.

Of the 197 initially identified children, a total of 132 were eligible to participate in the study. Based on the spirometry quality criteria applied, two acceptable spirometry curves were generated for 119 children. For each individual, the spirometric parameters of the maneuver that produced the best FVC+FEV_1_ or FEV_0.75_ values were selected. Figure 1 shows flow chart of the study population. As indicated above, FEV_0.75_ was preferred over FEV_1_ since FET was <1 s in 21.21% of the eligible population. In 104 children it was possible to determine FEV_1_, while in 118 FEV_0.75_ was recorded.

Table 1 shows the prenatal and neonatal variables based on the development of BPD. In total, 38.6% of the population developed BPD (22% mild, 16.6% moderate-to-severe). The multivariate model revealed that GA (0.456; CI95% 0.3–0.695; *p* < 0.001), hemodynamically significant PDA (19.491; CI95% 3.765–100.91; *p* < 0.001) and late sepsis (9.906; CI95% 1.947–50.392; *p* = 0.006) were independent risk factors for the development of BPD.

**Table 1:** Prenatal and neonatal variables stratified according to BPD diagnosis.

	**Without BPD**	**With BPD**	***p***
	**(*n* = 81)**	**(*n* = 51)**
Sex (male)	41 (50.6)	23 (45.1)	0.537
Premature rupture of membranes	32 (39.5)	21 (41.2)	0.849
Multiple gestation	37 (45.7)	28 (54.9)	0.302
Preeclampsia	12 (14.8)	4 (7.8)	0.283
Chorioamnionitis	13 (16)	15 (29.4)	0.067
Antenatal steroids	63 (77.8)	40 (78.4)	0.930
Delivery (caesarean section)	55 (67.9)	31 (60.8)	0.400
Gestational age (weeks)	30.9 ± 1.7	27.8 ± 1.7	<0.001
Birth Weight (grams)	1,327.7 ± 222.5	995.5 ± 210.2	<0.001
Intrauterine growth restriction	22 (27.2)	5 (9.8)	0.025
Apgar 1 min	8 (6–9)	5 (3–8)	<0.001
Apgar 5 min	9 (9–10)	9 (7–9)	<0.001
Intubation in delivery room	12 (14.8)	36 (70.6)	<0.001
CRIB index	1 (0–1)	2 (1–6)	<0.001
Surfactant administration	21 (25.9)	46 (90.2)	<0.001
2 or more doses of surfactant	3 (3.7)	15 (29.4)	<0.001
Early sepsis	2 (2.5)	9 (17.6)	0.003
Inotropes in the first week of life	7 (8.6)	17 (33.3)	<0.001
Invasive ventilation	19 (23.5)	45 (88.2)	<0.001
Invasive ventilation at day 7	7 (8.6)	26 (51)	<0.001
High frequency oscillatory ventilation	3 (3.7)	19 (37.3)	<0.001
Necrotizing entercolitis	0 (0)	6 (11.8)	0.003
Hemodynamically significant PDA	4 (4.9)	32 (62.7)	<0.001
Surgical PDA	0 (0)	13 (25.5)	<0.001
Late sepsis	5 (6.2)	23 (45.1)	<0.001
Hospital stay (days)	35 (28.5–45)	71 (54–87)	<0.001

*Results are expressed as frequencies for categorical variables, mean ± standard deviation for quantitative variables with normal distribution, and median (interquartile range) for variables that did not follow a normal distribution. BPD, brochopulmonary dysplasia; CRIB, clinical risk index for babies; PDA, patent ductus arteriosus*.

No association was found between lung function parameters and family history of allergy or asthma (33.6%), atopic dermatitis (31%), and parental smoking (44.2%). A history of recurrent wheezing (48.1%) was an independent risk factor for a decrease in FEV_0.75_ in the multivariate model (−0.515; CI95% −0.907 – −0.122; *p* = 0.011). Persistent recurrent wheezing at the time of the study, that can be considered as preschool asthma (16.8%), was associated with a decrease in FEV_0.75_ and FEV_0.75_/FVC (obstructive pattern), however these differences were not statistically significant (FEV_0.75_: −1.60 ± 1.79 SD vs. −0.96 ± 1.2 SD, *p* = 0.060; FEV_0.75_/FVC: −0.9 ± 0.77 SD vs. −0.57 ± 1.05 SD, *p* = 0.126), since the study was not powered for this analysis. For the same reason, BPD did not show a significant association with either the subsequent diagnosis of recurrent wheezing or its persistence (preschool asthma) at the time of the study.

**Figure 2:**
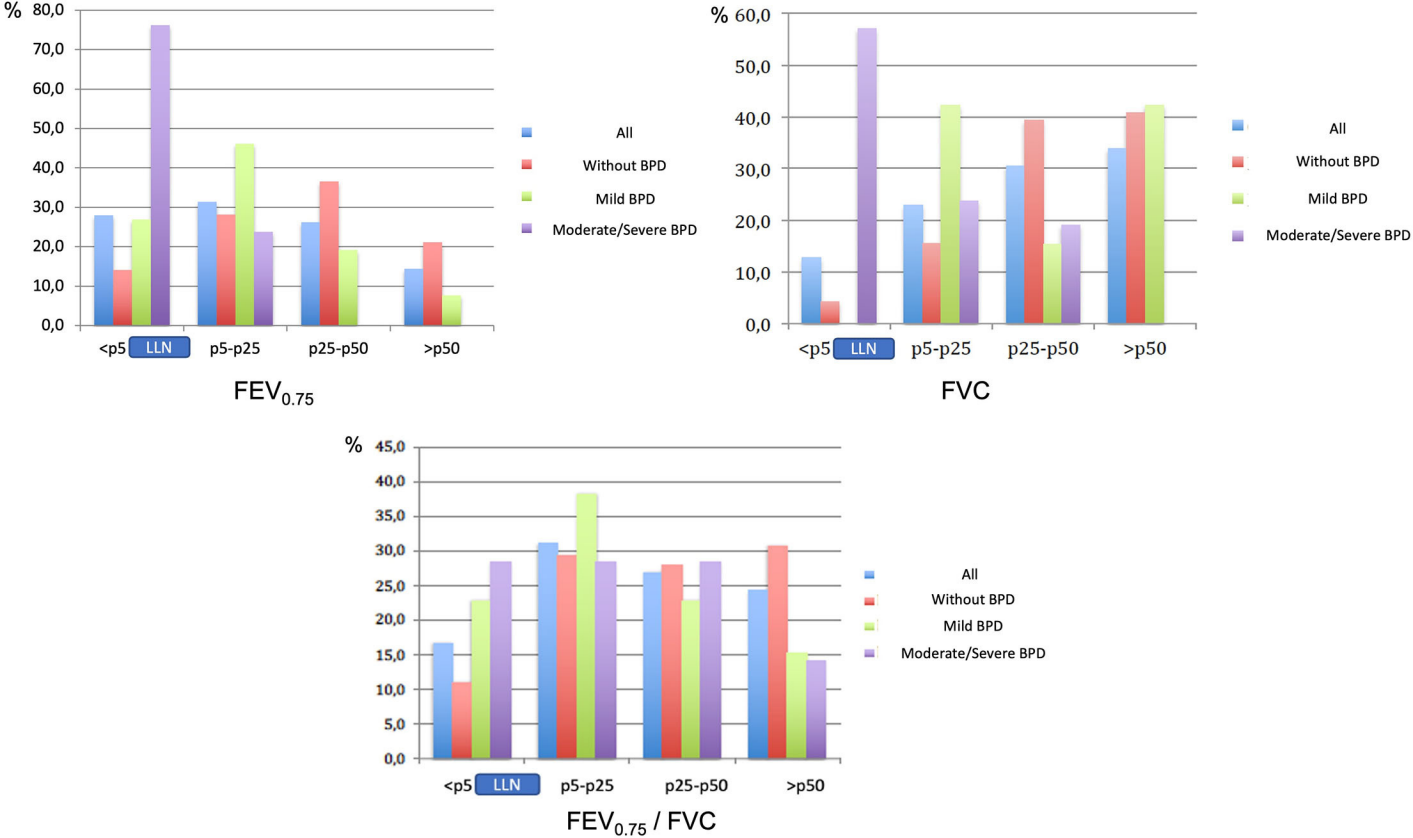
FEV0_.75_, FVC and FEV_0.75_/FVC: percentile distribution in BPD subgroups. FEV_0.75_ was beneath the LLN (5^th^ percentile) in a significant proportion of children in all subgroups, as follows: no BPD, 14.1%; mild BPD, 26.9%; moderate-to-severe BPD, 76.2%. FVC was beneath the LLN in 57.1% of children in the moderate-to-severe BPD subgroup. FEV_0.75_/FVC was beneath the LLN in a significant proportion in BPD subgroups, as follow, no BPD, 11, 3%; mild BPD, 23, 1%; moderate-to-severe BPD, 28.6%. BPD, bronchopulmonary dysplasia; FEV_0.75_, forced expiratory volume in the first 0.75 s; FVC, forced vital capacity; LLN, lower limit of normal (5^th^ percentile).

Table 2 shows lung function parameters relative to BPD diagnosis and severity. All groups showed a significant decrease in FEV_0.75_, FEF_75_, FEF_25−75_, FEV_1_/FVC and FEV_0.75_/FVC relative to the reference population, which may indicate a typical obstructive pattern. FVC was only significantly reduced in patients with a diagnosis of moderate-to-severe BPD, while FEV_1_ was significantly reduced in patients with BPD of any severity, with lower values observed for moderate-to-severe BPD. Figure 2 shows the proportion of children with FEV_0.75_, FVC and FEV_0.75_/FVC below the LLN. In a significant proportion in all groups, but in particular in those with moderate-to-severe BPD, FEV_0.75_ was below the LLN. Among children without BPD, 14.1% had a FEV_0.75_ below the LLN. This proportion increased to 26.9% among children with mild BPD and 76.2% among those with moderate-to-severe BPD. FVC was below the LLN in a significant proportion of children only in the subgroup with moderate-to-severe BPD. The percentile distribution for the entire population and data on other parameters related to BPD diagnosis and severity are provided in the Supplementary Material.

**Table 2:** Lung function parameters in children age 4–6 years according to BPD diagnosis.

	**No BPD**	**BPD**	**Mild BPD**	**Moderate/Severe BPD**
FVC	(*n* = 72) −0.18 ± 1.00 (−0.42–0.05) *p* = 0.128	(*n* = 47) −0.99 ± 1.24 (−1.36– −0.63) *p* < 0.001	(*n* = 26) −0.32 ± 0.90 (−0.69–0.04) *p* = 0.081	(*n* = 21) −1.82 ± 1.12 (−2.33– −1.31) *p* = 0.020
FEV_1_	(*n* = 64) −0.24 ± 1.03 (−0.50–0.02) *p* = 0.068	(*n* = 40) −1.32 ± 1.02 (−1.65– −0.99) *p* < 0.001	(*n* = 24) −0.83 ± 0.82 (−1.18– −0.48) *p* < 0.001	(*n* = 16) −2.06 ± 0.84 (−2.51– −1.61) *p* < 0.001
FEV_0.75_	(*n* = 71) −0.55 ± 1.12 (−0.82– −0.28) *p* < 0.001	(*n* = 47) −1.83 ± 1.22 (−2.19– −1.47) *p* < 0.001	(*n* = 26) −1.18 ± 0.80 (−1.50– −0.86) *p* < 0.001	(*n* = 21) −2.63 ± 1.18 (−3.16– −2.09) *p* < 0.001
FEF_75_	(*n* = 72) −0.33 ± 1.01 (−0.57– −0.10) *p* = 0.007	(*n* = 47) −1.00 ± 1.01 (−1.30– −0.71) *p* < 0.001	(*n* = 26) −0.95 ± 0.85 (−1.29– −0.61) *p* < 0.001	(*n* = 21) −1.08 ± 1.19 (−1.63– −0.54) *p* = 0.001
FEF_25−75_	(*n* = 72) −0.91 ± 0.99 (−1.14– −0.68) *p* < 0.001	(*n* = 47) −1.71 ± 0.89 (−1.97– −1.45) *p* < 0.001	(*n* = 26) −1.42 ± 0.75 (−1.72– −1.12) *p* < 0.001	(*n* = 21) −2.06 ± 0.94 (−2.49– −1.63) *p* < 0.001
FEV_1_/FVC	(*n* = 64) −0.44 ± 1.03 (−0.70– −0.18) *p* = 0.001	(*n* = 40) −0.86 ± 1.02 (−1.19– −0.53) *p* < 0.001	(*n* = 24) −0.92 ± 0.93 (−1.31– −0.52) *p* < 0.001	(*n* = 16) −0.78 ± 1.17 (−1.40– −0.15) *p* = 0.018
FEV_0.75_/FVC	(*n* = 71) −0.45 ± 1.05 (−0.70– −0.20) *p* = 0.001	(*n* = 47) −0.88 ± 0.91 (−1.15– −0.62) *P* < 0.001	(*n* = 26) −0.92 ± 0.81 (−1.25– −0.60) *p* < 0.001	(*n* = 21) −0.83 ± 1.04 (−1.31– −0.36) *p* = 0.002

*Results are expressed as the mean ± standard deviation z-score. The 95% confidence interval and the level of significance are indicated relative to the GLI2012 reference population. BPD, bronchopulmonary dysplasia; FVC, forced vital capacity; FEV_1_, forced expiratory volume in the first second; FEV_0.75_, forced expiratory volume in the first 0.75 seconds; FEF_75_, forced expiratory flow at 75% of FVC; FEF_25−75_, forced expiratory flow between 25 and 75% of FVC*.

As shown in Table 3, BPD (28 days) was associated with a significant decrease in all spirometry parameters at preschool age. When this analysis was performed for children diagnosed with BPD at 36 weeks (moderate-to-severe), the decrease in obstructive pattern parameters was more pronounced than in the total BPD group. This effect was particularly evident for FEV_0.75_ (−2.63 SD) and FEF_25−75_ (−2.06 SD).

**Table 3:** Lung function parameters in children age 4–6 years according to BPD diagnosis (28 days and 36 weeks).

**BPD 28 days**	**Without BPD**	**BPD**	***p***
FVC	−0.18 ± 1.00 (*n* = 72)	−0.99 ± 1.25 (*n* = 47)	<0.001
FEV_1_	−0.24 ± 1.03 (*n* = 64)	−1.32 ± 1.02 (*n* = 40)	<0.001
FEV_0.75_	−0.55 ± 1.13 (*n* = 71)	−1.83 ± 1.22 (*n* = 47)	<0.001
FEF_75_	−0.33 ± 1.01 (*n* = 72)	−1.00 ± 1.01 (*n* = 47)	0.001
FEF_25−75_	−0.91 ± 0.99 (*n* = 72)	−1.71 ± 0.89 (*n* = 47)	0.008
FEV_1_/FVC	−0.44 ± 1.03 (*n* = 64)	−0.86 ± 1.02 (*n* = 40)	0.045
FEV_0.75_/FVC	−0.45 ± 1.05 (*n* = 71)	−0.88 ± 0.91 (*n* = 47)	0.023
**BPD 36 weeks**	**Without BPD**	**BPD**	***p***
FVC	−0.22 ± 0.97 (*n* = 98)	−1.82 ± 1.12 (*n* = 21)	<0.001
FEV1	−0.40 ± 1.01 (*n* = 88)	−2.06 ± 0.84 (*n* = 16)	<0.001
FEV_0.75_	−0.72 ± 1.08 (*n* = 97)	−2.63 ± 1.18 (*n* = 21)	<0.001
FEF_75_	−0.50 ± 1.00 (*n* = 98)	−1.08 ± 1.20 (*n* = 21)	0.021
FEF_25−75_	−1.04 ± 0.95 (*n* = 98)	−2.06 ± 0.94 (*n* = 21)	<0.001
FEV_1_/FVC	−0.57 ± 1.02 (*n* = 88)	−0.78 ± 1.17 (*n* = 16)	0.467
FEV_0.75_/FVC	−0.58 ± 1.01 (*n* = 97)	−0.83 ± 1.04 (*n* = 21)	0.301
	**Without BPD**	**Mild BPD**	***p***
FVC	−0.18 ± 1.00 (*n* = 72)	−0.32 ± 0.90 (*n* = 26)	0.534
FEV_1_	−0.24 ± 1.03 (*n* = 64)	−0.83 ± 0.83 (*n* = 24)	0.013
FEV_0.75_	−0.55 ± 1.13 (*n* = 71)	−1.18 ± 0.80 (*n* = 26)	0.010
FEF_75_	−0.33 ± 1.01 (*n* = 72)	−0.95 ± 0.85 (*n* = 26)	0.007
FEF_25−75_	−0.91 ± 0.99 (*n* = 72)	−1.42 ± 0.75 (*n* = 26)	0.018
FEV_1_/FVC	−0.44 ± 1.03 (*n* = 64)	−0.92 ± 0.93 (*n* = 24)	0.052
FEV_0.75_/FVC	−0.45 ± 1.05 (*n* = 71)	−0.92 ± 0.81 (*n* = 26)	0.040

*Results are expressed as the mean ± standard deviation z-score. BPD, bronchopulmonary dysplasia; FVC, forced vital capacity; FEV_1_, forced expiratory volume in the first second; FEV_0.75_, forced expiratory volume in the first 0.75 seconds; FEF_75_, forced expiratory flow at 75% of FVC; FEF_25−75_, forced expiratory flow between 25 and 75% of FVC*.

**Figure 3:**
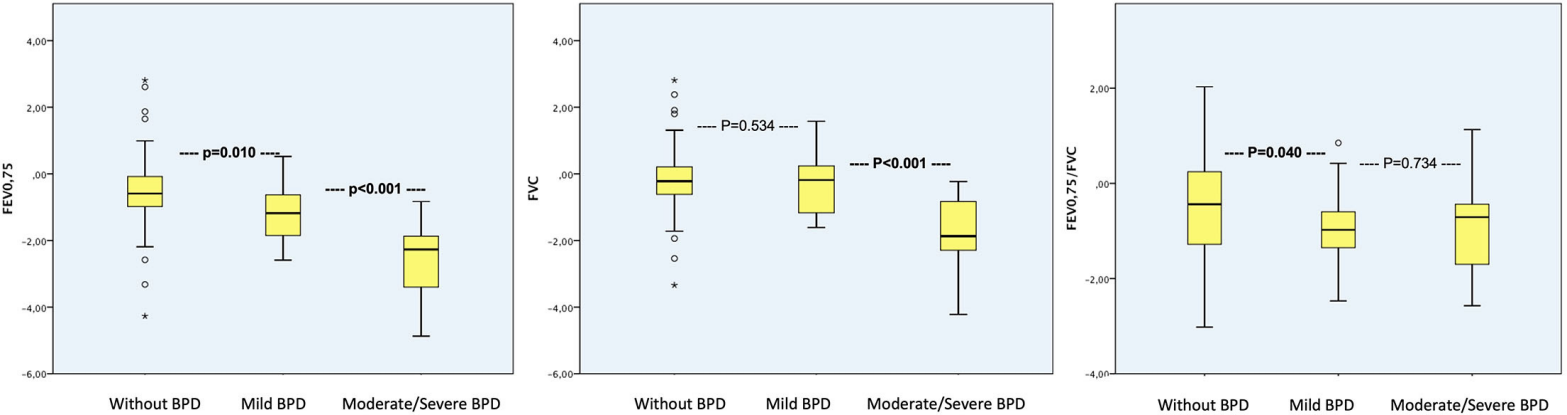
FEV_0.75_, FVC and FEV_0.75_/FVC in each BPD subgroup. Data are presented as the mean and standard deviation. FEV_0.75_, but not FVC, was significantly reduced in the mild BPD vs. the no BPD subgroup. Both FEV_0.75_ and FVC were significantly reduced in the moderate-to-severe vs. the no BPD subgroup. FEV_0.75_/FVC was significantly reduced in BPD subgroups vs. the no BPD subgroup. FEV_0.75_/FVC did not show statistically significant difference between mild and moderate-to -severe BPD. BPD, bronchopulmonary dysplasia; FEV_0.75_, forced expiratory volume in the first 0.75 s; FVC, forced vital capacity.

Figure 3 shows the differences in FEV_0.75_, FVC and FEV_0.75_/FVC between the different subgroups. Greater lung function impairment as evidenced by a decreased in FEV_0.75_ at age 4–6 years was observed in children with mild BPD than those without, but no differences in FVC were observed between these two groups. By contrast, FVC was significantly lower in children with moderate-to-severe BPD than in those without. FEV_0.75_/FVC was significantly reduced in BPD subgroups vs. the no BPD subgroup. FEV_0.75_/FVC did not show statistically significant difference between mild and moderate-to-severe BPD.

In the multivariate model moderate-to-severe BPD was the independent risk factor most closely associated with reduced FEV_0.75_ (B = −1.488, CI95% −2.077– −0.899; *p* < 0.001), followed by GA (B = 0.106, CI95% 0.010–0.202; *p* = 0.031) or recurrent wheezing (B = −0.515, CI95% −0.907– −0.122; p = 0.011). The only independent risk factor for reduced FVC was a history of moderate-to-severe BPD (B = −1.492, CI95% −1.980– −1.004; *p* < 0.001).

## Discussion

BPD is a disease that is arbitrarily defined by its treatment rather than its pathophysiology or clinical findings (19). This fact has conditioned multiple attempts to establish the definition that best predicts the long-term respiratory outcome in this population (20). The NIH 2001 consensus definition (7) took the important step of including a BPD severity scale, in contrast to the Sheenan definition (6), and subsequent studies demonstrated that infants with severe BPD have higher rates of mortality and adverse outcomes after discharge (21). However, the definition of severe BPD was overly broad, and combined infants receiving more than 30% oxygen via low flow nasal cannula with those receiving invasive ventilation. The definition proposed by the NICHD workshop in 2018 (9) reserved grade three for infants receiving invasive ventilation or non-invasive ventilation / nasal cannula >3 l/min in addition to oxygen ≥30%. This definition also assigned a diagnosis of BDP to preterm infants who die before 36 weeks PMA due to persistent parenchymal lung disease and respiratory failure that cannot be attributable to other neonatal morbidities. However, this definition eliminated the requirement for 28 days of oxygen therapy before 36 weeks PMA, as also occurs in Jensen's evidence-based definition (10). Indeed, the latter even eliminated from the definition the need for oxygen, which had previously formed the basis of the definition of BPD. It is very likely that the modifications introduced by the NICHD workshop and by Jensen improve the predictive capacity of the definition of BPD, although validation based exclusively on early respiratory outcome may be insufficient, and may need to also include assessment of lung function throughout childhood.

In the present study we sought to evaluate the utility of the NIH consensus definition of BPD (7) to predict lung function in preschool age children (4–6 years), as measured using forced incentive spirometry. We investigated whether basing the definition on the need for oxygen and the requirement of oxygen therapy for 28 days prior to 36 weeks PMA remain relevant to the definition of BPD.

Spirometry is one of the most commonly used techniques for evaluation of lung function, can be used in children of 3 years and older, and has been proposed as an appropriate means of assessing lung function in children and adults with a history of prematurity (22–25). However, few studies have used this technique in preschool age children (26). The results of a previous study (15) that analyzed the suitability of this technique for assessment of lung function in healthy preschoolers support widespread application of the GLI-2012 reference equations. The GLI-2021 group proposes using FEV_0.75_ rather than FEV_1_ in preschool children, as not all children of this age achieve a FET >1 second, the FEV_1_/FVC ratio is very close to one, and FEV_0.75_ more reliably reflects central and peripheral airway alterations. For this reason, we considered FEV_0.75_ more appropriate than FEV_1_ for the evaluation of lung function by forced spirometry.

Overall, our study cohort showed a significant reduction in all lung function parameters relative to GLI2012 standard values. More significant changes were observed for FEV_0.75_ and FEF_25−75_, parameters that reflect the predominance of an obstructive pattern linked to prematurity. In fact, analysis of pulmonary function parameters in the subgroup without BPD revealed significant reductions only in the parameters that reflect an obstructive pattern, in line with the findings of other studies (22, 23, 25). This alteration was considerably more significant among those with a diagnosis of BPD.

From a clinical point of view, the LLN, defined as the 5^th^ percentile or a z-score of −1.64, is adequate when establishing normality values, even more than 80% of the predicted value (27). In all subgroups in our cohort, values for FEV_0.75_ and other obstructive pattern parameters were below the 5^th^ percentile for a significant percentage of children. Among children without BPD or with mild BPD 14.1 and 26.9%, respectively, were below the 5^th^ percentile for FEV_0.75_. This percentage increased to 76.2% in the case of children with moderate-to-severe BPD. The percentage of children with FVC below the 5^th^ percentile was <5% among those without BPD or with mild BPD. However, FVC was below the 5^th^ percentile in more than 50% of those diagnosed with moderate-to-severe BPD, confirming that alteration of this parameter was exclusive to this group. The EPICure study (28) reported a similar lung function in BPD patients. In children with BPD, impairment of expiration may be due not only to airway obstruction, but also to a decrease in lung volumes. The “new BPD” corresponds to an arrest of alveolar growth, and therefore it is logical that restrictive or mixed defects in lung function will be observed. The pattern observed in children with moderate-to-severe BPD clearly differed from that seen in the other subgroups. Moreover, children with a diagnosis of mild BPD in the neonatal period presented a pattern similar to that observed in those without BPD, exhibiting no restrictive alterations but greater alterations in FEV_0.75_. This greater impairment of lung function in mild BPD does not support exclusion from the definition of BPD of the requirement of 28 days of oxygen therapy before 36 weeks PMA. Recently, Chang et al. (26) reported a significant negative association between BPD severity and FEV_1_, FVC, and FEF_25−75_, although flow characteristics in mild BPD did not differ significantly from those of preterm children without BPD and full-term controls. It is not clear whether the more pronounced obstructive pattern observed in our cohort, characterized by a significant reduction in FEV_0.75_ and FEV_0.75_/FVC, will improve as these patients age. Given the absence of FVC involvement, this subgroup could be equated with that of children without BPD if improvement occurs over the following years.

Reduced small airway patency, as assessed by FEF_25−75_, has been reported during different stages of infancy (26, 28–31). In our study, all groups showed a reduction of FEF_25−75_ relative to the reference population. However, BPD was associated with a greater alteration in small airway parameter, especially in patients with moderate-to-severe BPD.

BPD is the result of complex interactions between altered alveolar and vascular development, lesions induced by antenatal and postnatal pathogenic factors, and reparative processes in the lungs (1). In our population, GA, hemodynamically significant PDA, and late sepsis were independent risk factors for BPD. Given that some of these conditions and other antenatal and neonatal evolution variables were associated with altered lung function in the univariate analysis, we conducted a subsequent multivariate analysis, which verified that moderate-to-severe BPD is the most significant independent risk factor for lung function impairment at 4–6 years of age. BPD was the only variable associated with alterations in FVC.

Wheeze is a common diagnosis in preschool children. The prevalence presents large variations, from 7.7 to 55% in different cohorts (32). In our cohort about 50% had a history of recurrent wheezing in early childhood and in 16.8% it persisted at the time of the study. Recurrent wheezing at this age is related to viral infections and its progression to asthma is conditioned by different factors. The factors most strongly associated with progression to asthma are wheeze frequency and severity, atopy, maternal asthma severity and probably prematurity. Nevertheless, it is not clear to what extent a history of BPD involves and additional risk. Recently, the EPIPAGE cohort study (33) shows that preschool wheeze and BPD were the only independent variables associated with FEV_1_ impairment in adolescents, as our study shows in preschool age. Pérez Tarazona et al. (34), in a systematic review found that the prevalence of asthma was higher in children and adolescents with a history of prematurity and BPD compared with those who did not develop BPD. However, in only one of the 17 studies included, this difference reaches statistical significance.

A strength of the present study is the demonstration that incentive spirometry is adequate to analyse lung function in preschool children with a history of prematurity, in strict accordance with international quality criteria for the performance of this technique. Another strength is the multivariate analysis performed, which shows that moderate-to-severe BPD is a risk factor for lung function impairment at preschool age, independent of other neonatal variables such as gestational age, birthweight, or neonatal morbidities.

This study has several limitations. First, we applied a retrospective observational design, which is common in lung function studies in preterm newborns. Second, children with significant neurological involvement were excluded due to technical difficulties that arise when performing spirometry in these children. This decreased the sample size of the subgroup with severe BPD, which was consequently grouped with the moderate BPD subgroup for analyses. However, this is considered a minor limitation, given that most studies analyzing BPD as a primary outcome consider BPD at 36 weeks PMA.

In conclusion, the NIH consensus definition is adequately predictive of lung function measured by spirometry at 4–6 years of age. Children with a history of mild BPD present a greater degree of impairment of lung function parameters than those without, but do not present FVC reduction at the age of 4–6 years. Children with diagnosis of moderate-to-severe BPD exhibit a mixed pattern, being the only form associated with FVC impairment at this age. Future attempts to validate different BPD definitions should consider long-term respiratory outcomes. This population is at risk of not achieving full airway growth potential and developing chronic obstructive pulmonary disease in adulthood.

## Data Availability Statement

The raw data supporting the conclusions of this article will be made available by the authors, without undue reservation.

## Ethics Statement

The studies involving human participants were reviewed and approved by Ethics and Research Committee of Aragón, CEICA (PI15/002). Written informed consent to participate in this study was provided by the participants' legal guardian/next of kin.

## Author Contributions

SR, CM, and CR conceived the study. SR, CM, CR, and JG-I contributed to the design of the clinical work. CR, JG-I, and CM performed the incentive spirometry studies. SR and CR were responsible for data acquisition. SR and AM performed the statistical analyses. MC and MS critically reviewed the manuscript. All authors participated in the interpretation of the study results, as well as the drafting and revision of the manuscript, and all approved the final version of the manuscript prior to submission.

## Funding

This study was partially financed with funds from the IDIS research group C012 (Santiago de Compostela) and Miguel Servet Biomedical Foundation (Zaragoza).

## Conflict of Interest

The authors declare that the research was conducted in the absence of any commercial or financial relationships that could be construed as a potential conflict of interest.

## Publisher's Note

All claims expressed in this article are solely those of the authors and do not necessarily represent those of their affiliated organizations, or those of the publisher, the editors and the reviewers. Any product that may be evaluated in this article, or claim that may be made by its manufacturer, is not guaranteed or endorsed by the publisher.
