# Supplementary material for: The Consensus Definition of Bronchopulmonary Dysplasia Is an Adequate Predictor of Lung Function at Preschool Age
Source: Front Pediatr. 2022 Feb 4;10:830035. doi: 10.3389/fped.2022.830035 (PMC8854776; doi:10.3389/fped.2022.830035)
Supplement: Supplementary file 1 [file Data_Sheet_1.pdf]

SUPPLEMENTARY MATERIAL

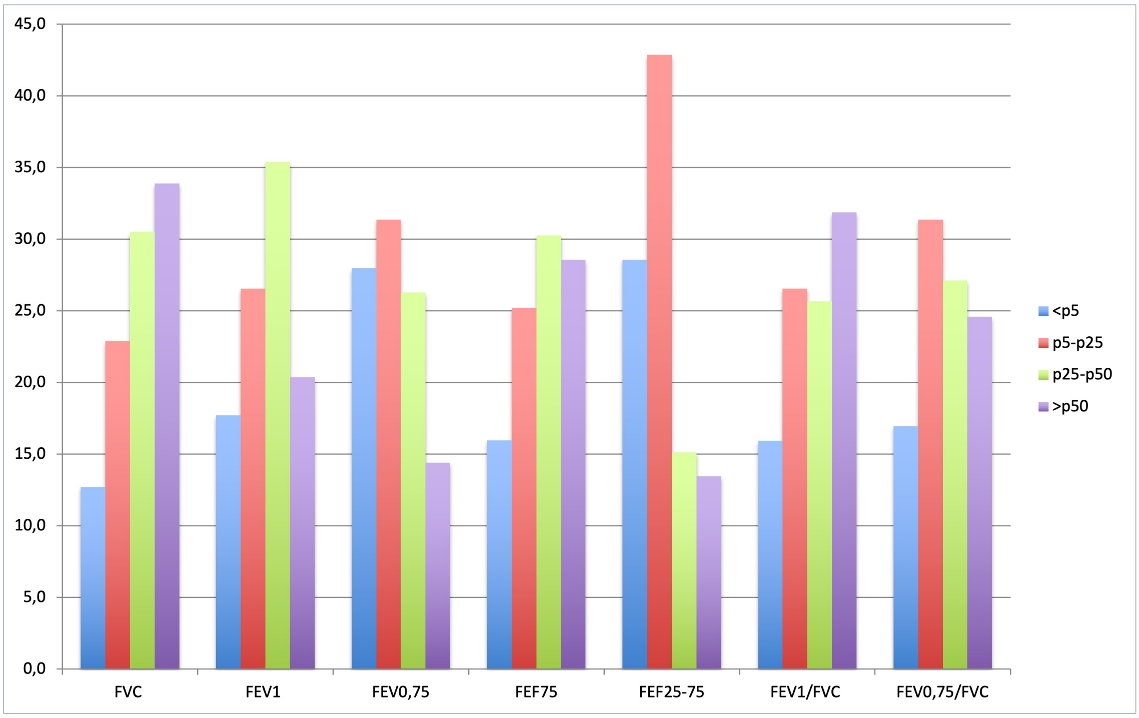

Figure S1. Percentile distribution of the study population for the spirometric parameters analyzed. A high proportion of subjects were beneath de LLN (5th percentile) for all parameters, being more prominent for $FEV_{0.75}$ (28%) and FEF25-75 (28.6%). FVC, forced vital capacity; FEV1, forced expiratory volume in the first second; $FEV_{0.75}$, forced expiratory volumen in the first 0.75 seconds; FEF_{75}, forced expiratory flow at 75% of FVC; $FEF_{25\text{-}75}$, forced expiratory flow between 25% and 75% of FVC.

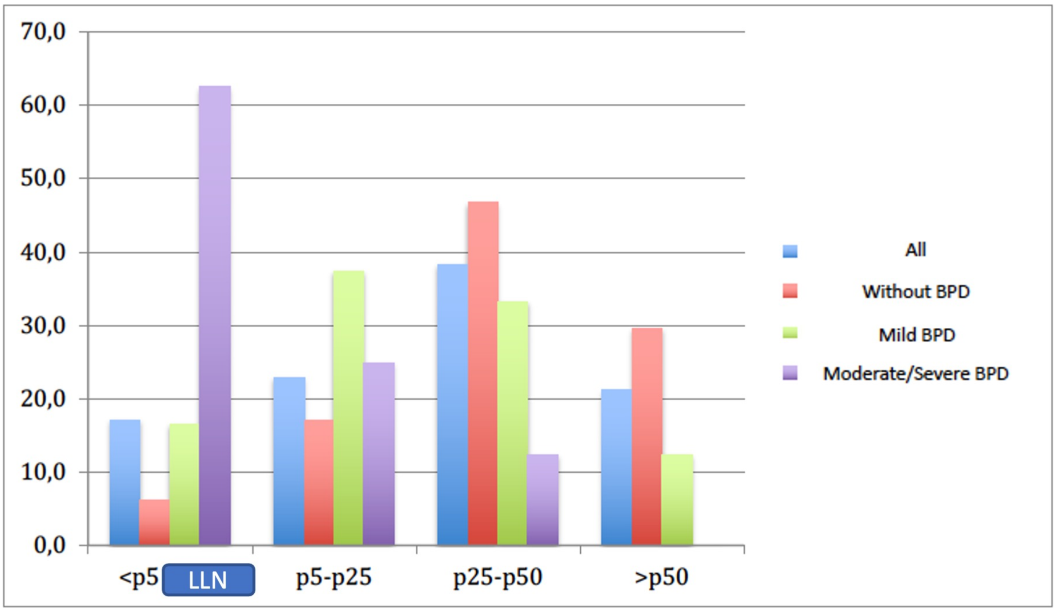

Figure S2. FEV_1: percentile distribution in BPD subgroups. FEV_1 was beneath the LLN (5th percentile) in a significant proportion of children in BPD subgroups, as follows: no BPD, 6.3%; mild BPD, 16.7%; moderate-to-severe BPD, 62.5%. BPD, bronchopulmonary dysplasia; FEV_1, forced expiratory volume in the first second; LLN, lower limit of normal (5th percentile).

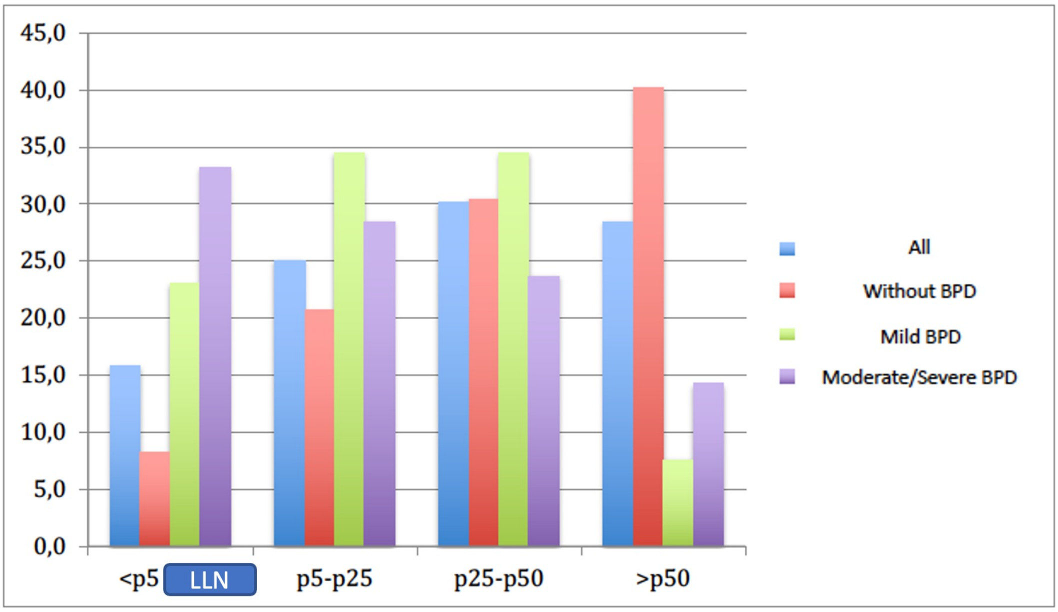

Figure S3. FEF_{75}: percentile distribution in BPD subgroups. FEF_{75} was beneath the LLN (5th percentile) in a significant proportion of children in BPD subgroups, as follows: no BPD, 8.3%; mild BPD, 23.1%; moderate-to-severe BPD, 33.3%. BPD, bronchopulmonary dysplasia; FEF_{75}, forced expiratory flow at 75% of FVC; LLN, lower limit of normal (5th percentile).

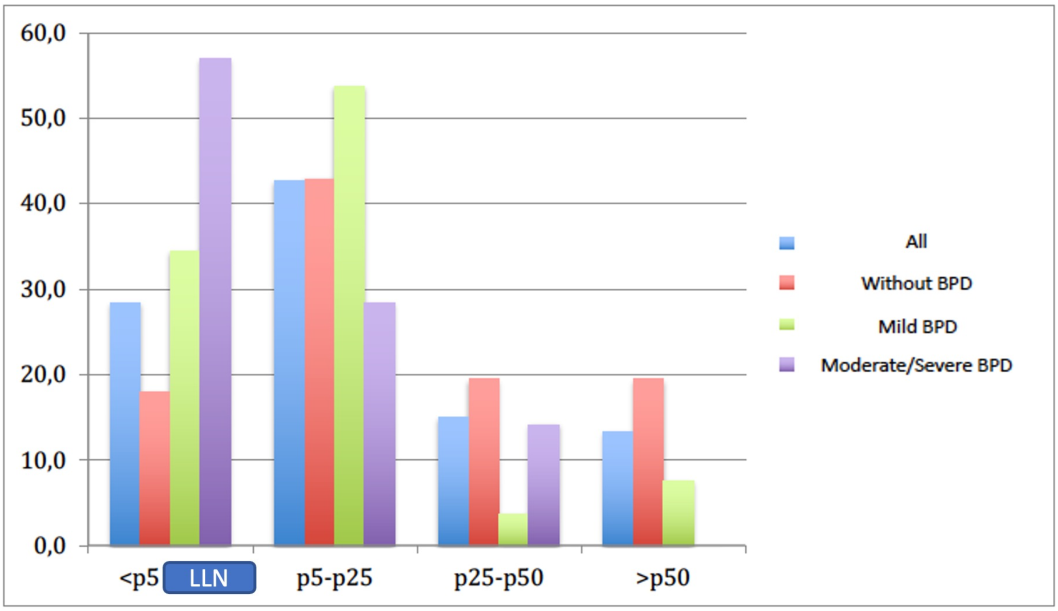

Figure S4. FEF_{25-75}: percentile distribution in BPD subgroups. FEF_{25-75} was beneath the LLN (5th percentile) in a significant proportion of children in BPD subgroups, as follows: no BPD, 18.1%; mild BPD, 34.6%; moderate-to-severe BPD, 57.1%. BPD, bronchopulmonary dysplasia; FEF_{25-75}, forced expiratory flow between 25% and 75% of FVC; LLN, lower limit of normal (5th percentile).

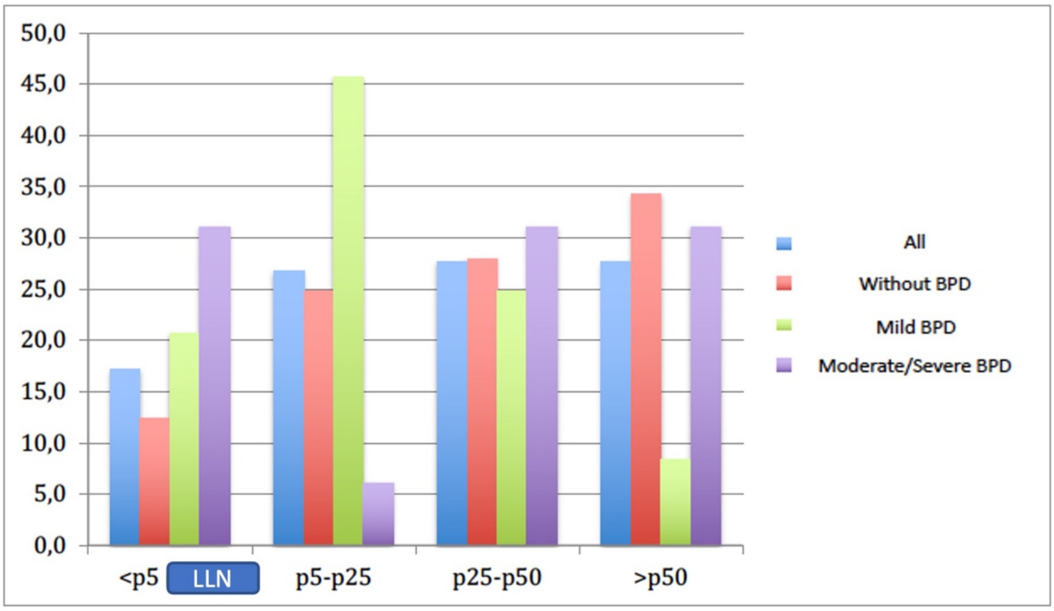

Figure S5. FEV_1/FVC: percentile distribution in BPD subgroups. FEV_1/FVC was beneath the LLN (5th percentile) in a significant proportion of children in BPD subgroups, as follows: no BPD, 12.5%; mild BPD, 20.8%; moderate-to-severe BPD, 31.3%. BPD, bronchopulmonary dysplasia; FEV_1/FVC, ratio between the forced expiratory volumen in the first second and forced vital capacity; LLN, lower limit of normal (5th percentile).
